# Clinical and immunological evaluation of cat‐allergic asthmatics living with or without a cat

**DOI:** 10.1111/cea.14024

**Published:** 2021-10-08

**Authors:** Erik R. Wambre, Mary Farrington, Veronique Bajzik, Hannah A. DeBerg, Marcella Ruddy, Michelle DeVeaux, Pretty Meier, David Robinson, Matt Cantor, Chengrui Huang, Jamie M. Orengo, Claire Q. Wang, Allen Radin

**Affiliations:** ^1^ Benaroya Research Institute at Virginia Mason Medical Center Seattle Washington USA; ^2^ Virginia Mason Medical Center Seattle Washington USA; ^3^ Regeneron Pharmaceuticals Tarrytown New York USA; ^4^ Koneksa Health New York New York USA

**Keywords:** basophil activation, cat allergy, Fel d 1, immunoglobulins, Th2A cells

## Abstract

**Background:**

Characterising the clinical and immunological impact of daily cat exposure in cat‐allergic subjects with asthma who live with cats (WC) and those who do not (WoC) may provide understanding of the drivers of the allergic response.

**Methods:**

Clinical and immunological characteristics (skin prick test, spirometry, symptom assessments, immunological markers) were compared between asthmatic subjects WC (*n* = 10) and WoC (*n* = 9).

**Results:**

WC subjects had greater use of long‐acting beta agonists (*p* < .05) and high‐potency corticosteroids. No differences were observed in lung function, nasal and ocular symptoms, or asthma control between the groups. Cat dander‐ and Fel d 1‐specific IgG_4_ concentrations were higher in WC than WoC subjects (both *p* < .05). Total IgE and cat dander‐, Fel d 1‐ and Fel d 7‐specific IgE concentrations were similar, but Fel d 4‐sIgE was higher in WC subjects (*p* < .05) versus WoC. Basophil sensitivity to cat dander extract and Fel d 1 was lower in WC versus WoC subjects (*p* < .05) and correlated with higher IgG_4_ concentrations (*r* = 0.63; *p* = .009). Fel d 1‐specific CD4+ T‐cell responses polarised toward Th2A responses in WC versus WoC subjects; Fel d 1‐specific IgE correlated with surface expression of CRTH2 and CD200R (both *p* ≤ .05).

**Conclusion:**

Immunological differences observed in WC versus WoC did not reflect clinical tolerance with natural cat exposure. The ability to live with a cat despite allergy could be driven by higher preventative medication use. This study may support design of novel therapeutics for allergy management.

## Graphical abstract

Exploratory study characterizing the impact of daily cat exposure in cat‐allergic subjects living with/without cats (WC/WoC). Cat dander‐ and Fel d 1‐specific IgG4 concentrations were higher in WC versus WoC subjects (*p* < .05); lower basophil sensitivity to cat dander extract/Fel d 1 in WC versus WoC subjects (*p* < .05) correlated with higher IgG4 concentrations (*r* = 0.63; *p *= .009). Immunological differences not reflected in clinical tolerance with cat exposure. Fel d 1, major cat allergen; Ig, immunoglobulin; WC, with cat; WoC, without cat.

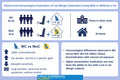

**KEYMESSAGES**

- Cat‐allergic asthmatics who live with cats have significantly higher medication requirements to control symptoms compared with cat‐allergic asthmatics who do not live with cats.
- Basophils are less sensitive to Fel d 1 stimulation in those who live with cat, which may be due to higher serum concentrations of allergen‐specific IgG4.
- Fel d 1‐specific Th2A responses are higher in those who live with cats relative to those who do not, and correlate with Fel d 1 sIgE levels.

## 1 INTRODUCTION

Estimates suggest that 25% to 38% of the US population live with cats^1^, ^2^ including individuals with a cat allergy, which is present in ~12% of the US population ≥6 years of age.^3^ Specific IgE to the major cat allergen, Fel d 1, is present in up to 95% of cat‐allergic individuals.^4^ Minor allergens are less frequently present, but have also been implicated in the allergic response.

Treatment of cat allergy is symptomatically driven, but allergen‐specific immunotherapy (AIT) is an option for those who do not respond to symptomatic therapy. While AIT is based on stimulating IgG production and/or targeting T‐cell responses^5^ and is considered to be effective with some allergens, it can require treatment for up to 5 years, may be associated with adverse events, and has equivocal clinical efficacy for cat allergy.^6^

Natural cat exposure has been implicated in conveying partial clinical and immunological tolerance, potentially mediated by IgG.^7^ However, the role of IgG in cat allergy is complex; although some studies showed that accumulation of IgG_4_ by chronic activation of the Th2 response may contribute to low level tolerance,^8^ other AIT studies suggested that the blocking potency of native IgG may be heterogeneous and not efficient.^9^ An explanation could be that both quantity and specificity of IgG may be important factors in attenuating the allergic response, as high affinity allergen‐blocking monoclonal IgG antibodies reduced the allergic response in animal models and cat‐allergic subjects.^9^ Consequently, evaluation of management strategies for cat allergy relies on understanding baseline clinical and immunological drivers of the allergic response. Since such an understanding is critical in the design of studies for evaluating new therapeutic approaches, the objective of this study was to characterise the impact of daily cat exposure by exploring relationships between clinical symptoms and immune mechanisms in cat allergic individuals with asthma who live with cats and those who do not live with cats.

## 2 METHODS

### 2.1 Study design and subjects

This exploratory, observational study compared clinical and immunological characteristics between asthmatic subjects diagnosed with cat allergy who live with cats (WC) and those who live without cats (WoC) (1:1 ratio). For this study, WC was defined as living with close, regular contact with a cat for at least 1 year, and WoC were subjects who generally avoid cats and have little or no cat exposure. The study consisted of three in‐clinic visits (screening, Day 1, and Day 28) and at‐home completion of diaries and assessments as described below for the specific outcomes. The study received approval from the Benaroya Research Institute (BRI) Institutional Review Board (IRB07109‐519), and all subjects provided written informed consent and Health Insurance Portability and Accountability Act authorisation prior to participation.

Subjects were identified from the BRI General Allergy Registry Biorepository and Virginia Mason allergy clinic, and were recruited by IRB approved in‐clinic flyers, phone and email requests. For inclusion, subjects were required to be adults 18–65 years old, inclusive, with a history of cat‐triggered allergic rhinitis and asthma for at least 2 years that had been diagnosed by an allergist and subsequently confirmed by the principal investigator. Subjects were also required to demonstrate immunological responses that included a skin prick test (SPT) mean wheal diameter ≥5 mm than negative control to standardised cat hair extract, and IgE ≥0.35 kU/L to cat dander. Additional inclusion criteria were the ability to stop antihistamine use for 5 days prior to each visit for SPT or basophil activation test (BAT), and possession of a vacuum that could accommodate an allergen collection adapter. Exclusion criteria were a history of cat AIT or any AIT within 6 months; concomitant dog allergy among subjects currently living with dog(s); significant mechanical nasal obstruction, or history of nasal or sinus surgery; a history of smoking within 6 months prior to screening; and use of systemic corticosteroids within 4 weeks prior to visit 2 (Day 1). While use of controller medications (leukotriene modifiers, long‐acting bronchodilators, and daily inhaled corticosteroids up to 1000 mcg Fluticasone equivalent) was allowed, doses >1000 mcg Fluticasone equivalent of inhaled corticosteroids or regular oral corticosteroid was cause for exclusion, as was use of biological agents (e.g., omalizumab, dupilumab, mepolizumab) within 6 months prior to screening.

### 2.2 Clinical measures

To determine allergen exposure, samples from each subject's mattress/bedroom were collected on Days 1 and 28 using the Indoor Biotechnologies DUSTREAM^TM^ Collector vacuum adapter, with analyses of the samples also conducted by Indoor Biotechnologies, Inc. (Charlottesville, VA); allergens assessed included Der p 1, Der f 1 (dust mites), Fel d 1 and Fel d 4 (cat), Can f 1 (dog), Mus m 1 (mouse), and Phl p 5 (timothy grass). Objective measures of clinical symptoms, assessed as described in the supplementary methods, were the forced expiratory volume in 1 s (FEV1, measured morning and evening daily during in‐home assessments and on Days 1 and 28 during in‐clinic visits) and the peak nasal inspiratory flow (PNIF; measured in‐clinic on Days 1 and 28).

Validated subject‐reported measures of clinical symptoms included the daily Total Nasal Symptom Score (TNSS),^10^ the Total Ocular Symptom Score (TOSS), the weekly Asthma Control Questionnaire (ACQ),^11^ and the Rhinoconjunctivitis Quality of Life Questionnaire (RQLQ) administered biweekly to assess health‐related quality of life.^12^ Details of these subject‐reported measures are described in the supplementary methods.

Subjects also completed daily medication e‐diaries, which were used to calculate a Daily Medication Score (DMS: 0 = none; 1 = antihistamine or topical nasal treatment; 2 = antihistamine *and* topical nasal treatment; and/or albuterol use (<5 puffs); 3 = low dose inhaled corticosteroid or leukotriene inhibitor; 4 = low dose inhaled corticosteroid *plus* leukotriene inhibitor or long‐acting beta agonist or albuterol use (5–8 puffs); 5 = high dose inhaled corticosteroid *plus* leukotriene inhibitor or long‐acting beta agonist or both; 6 = oral corticosteroid, or albuterol use >8 puffs). Consistent with recommendations for a single composite score,^13^, ^14^ the DMS was used as part of a composite Symptom and Medication Score (CSMS) as a measure of overall symptoms and consisted of TNSS+TOSS+DMS; the CSMS had a maximum score of 24 with higher scores indicative of a greater symptom burden.

### 2.3 Immunological and biomarker assessments

Imunological and biomarker assessments were conducted during the study visits on Days 1 and 28, and included SPT; serum sIgE, sIgG_4_, and total IgE assessments; blood eosinophil count, BAT; and cat allergen‐specific T‐cell analysis.

The SPT (Jubilant HollisterStier Allergy) was conducted via a standard protocol^15^ using allergen extracts and controls reported in the supplementary methods of the Online Respository.

Total IgE level, complete blood count with differential (eosinophil count), and serum allergen‐specific antibody testing using ImmunoCAP^®^ FEIA assays (ThermoFisher Scientific, Waltham, MA) were performed by Quest Diagnostics and included cat dander IgE (e1) and IgG_4_ (34909), *Dermatophagoides farinae* IgE (d1), *Dermatophagoides pteronyssinus* IgE (d2), timothy grass IgE (g6), dog dander IgE (e5A), and mouse urine IgE (e72). ImmunoCAP^®^ FEIA for serum IgE and IgG_4_ for Fel d 1, Fel d 4 and Fel d 7 were performed at Viracor Eurofins BioPharma Services (Lee's Summit, MO).

BATs were conducted within 6 h of blood draw on Days 1 and 28. Whole heparinised blood was stimulated for 30 min with anti‐IgE anti‐FceRI and a pool containing 8 distinct allergen extracts (grass, alder/birch, mould, and house dust mite mixes, cat, egg, milk, and walnut; Jubilant HollisterStier Allergy) as positive controls, media alone as a negative control, and high concentration of allergen (10,000 ng/ml) for cat extract, grass pollen mix, and a mixture of dust mite mix/dog extract and mouse extract (Jubilant HollisterStier Allergy), LoTox natural Fel d 1 and recombinant Fel d 4 (both from Indoor biotechnologies, Inc.), and Fel d 7, (kindly provided by Dr. Belinda Hales, Telethon Kids Institute). All stimuli were prepared in RPMI. Non‐activated and resting basophils were defined as CD123+HLADR‐CRTH2+CD3‐, in vitro activated basophils were evaluated based on CD203 and CD63 expression. Basophil sensitivity tests (BST), which measure the concentration required to elicit a basophil response, were performed for cat extract and Fel d 1 using a series of seven dilutions of allergen (10,000, 1000, 100, 10, 1, 0.1 and 0.01 ng/ml). Basophil allergen threshold sensitivity was determined using a dose response curve and expressed as the allergen concentration resulting in 50% of the maximum upregulation of CD63 expression (EC_50_).^16^ Basophil sensitisation curves were analysed using R software v 3.5.2,^17^ and Markov chain Monte Carlo simulation was performed using Stan.^18^ v 3.5.2.^17^

Cat allergen‐reactive CD4+ T cells were tracked using the CD154 and CD137 up‐regulation assay^19^, ^20^ as further described in the supplementary methods. T‐cell characterisation included assessment for Th2A cells, which represent a distinct subpopulation that contributes to induction of the allergic response.^21^

CD4+ T cells cultured for 14 days with specific immunodominant peptide were stained with corresponding phycoerythrin‐conjugated peptide‐MHCII‐tetramers (60 min, 37°C). Cells were then stimulated with 25 ng/ml phorbol 12‐myristate 13‐acetate and 1 μg/ml ionomycin in the presence of 10 µg/ml brefeldin‐A (4 h, 37°C, 5% CO_2_). Surface staining was followed by fixation/permeabilisation as per the manufacturer's protocol (eBioscience). Cells were stained (30 min, 4°C) with combinations of antibodies for IFN‐γ, IL‐17, and IL‐10 (all Biolegend), IL‐4 (eBioscience), and IL‐5 (Miltenyi Biotec), or corresponding isotype‐matched mAbs. Cells were washed and immediately analysed by flow cytometry.

### 2.4 Statistical analysis

No power calculations were considered to determine sample size. Furthermore, because of the variety of assessments, the enrolment goal was 10 WC subjects and 10 WoC subjects.

Between‐group comparisons were conducted using *t*‐tests or non‐parametric tests (Mann‐Whitney test) as appropriate, and are expressed as nominal *p*‐values. Within‐subject comparisons of in‐clinic versus at‐home spirometry were evaluated using a non‐parametric Wilcoxon matched‐pairs signed‐rank test. Spearman correlations explored associations between immunological markers; strength of the correlation was considered weak if the absolute value of the correlation coefficient (*r*) was <0.30, moderate if between 0.30 and 0.50, and strong if >0.50.^22^

## 3 RESULTS

### 3.1 Population and allergen exposure

The analysis included 10 asthmatic WC individuals and 9 WoC subjects; 1 WoC subject was excluded because of a prior history of cat AIT discovered after study completion. All subjects had a history of cat allergy‐induced allergic rhinitis and asthma symptoms of at least 5 years. Demographics were similar between the two groups except for dog ownership, which was reported by 4 WoC subjects and one WC subject (see Table S1). Of the WC subjects, 5 had 1 cat, 4 had 2 cats, and 1 had 10 cats, and while one subject had been living with a cat for 1.5 years, all other WC subjects had been living with cat(s) for ≥3 years (Table S2). All WC subjects reported that the cats were indoors >20 h per day.

Home cat allergen exposure, expressed as µg/g of dust collected, was significantly higher among WC subjects relative to WoC, as indicated by median levels of Fel d 1 (37.5 vs. 0.1 µg/g) and Fel d 4 (10.4 vs. 0 µg/g) in collected bedroom dust (both *p* < .05; Table S3). While exposure was negligible for Der p 1, Der f 1, Mus m 1, and Phl p 5, WoC subjects had significantly higher Can f 1 (1.7 vs. 0.04; *p* < .05) exposure (Table S3), likely reflecting the dog ownership reported by 4 of these subjects. The value of indoor allergen assessment was limited, however, by high variability between samples from the two time points.

### 3.2 Medication burden

**FIGURE 1:**
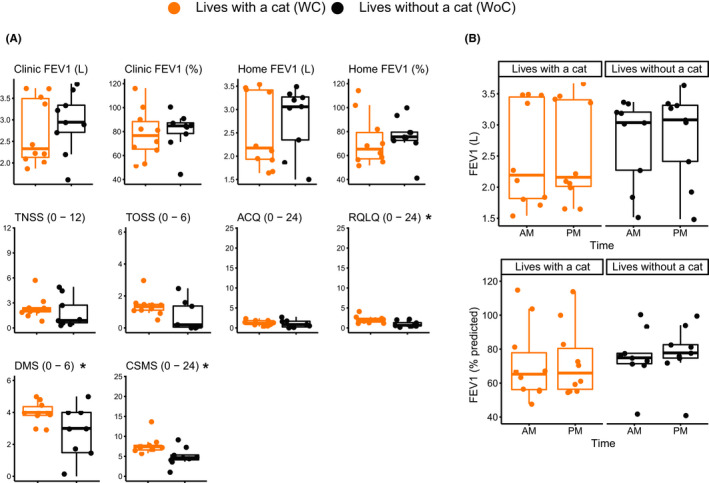
Clinical measures are generally similar in cat allergic subjects with or without a cat, and demonstrate robustness of measurement within subjects and over time. (A) Box plots comparing clinical measures in cat‐allergic asthmatics living with a cat to those living without a cat. At‐home FEV data reflect 28 days average of spirometry assessments. (B) Consistency of time‐dependent spirometry assessment of lung function. The solid line (−) is the mean, and ends of the “box” are the first and third quartiles, with the whiskers showing the maximum and minimum values; points outside the plots represent outliers. *p value <.05

The WC subjects had greater preventative medication requirements, including significantly higher long acting beta agonist use (*p* < .05) and generally used more potent corticosteroids at higher doses. The higher medication burden was further manifested by significantly higher mean scores on the DMS (4.0 ± 0.7 vs. 2.5 ± 1.8) and CSMS (7.8 ± 2.2 vs. 5.0) ± 2.3 (both *p* < .05). Asthma severity classification was higher among WC due to the increased requirement for preventative medications (Table S4). No significant differences were observed between the two groups in lung function including FEV1, FEV1% predicted, and in‐clinic PNIF (Figure 1A and Table S4). Similarly, there were no differences for nasal and ocular symptoms scores (TNSS, TOSS), or ACQ between the two groups. These similarities between the two groups, especially lung function, may be partly accounted for by the higher medication burden among WC subjects, since the medications likely ameliorated clinical symptoms. However, WC subjects reported higher scores on the RQLQ (mean 2.1 ± 0.9 vs 1.0 ± 0.8; *p* < .05) with a difference in score that exceeded the minimal clinically important difference indicating that this difference is clinically meaningful.

### 3.3 Lung function

Assessment of lung function showed high intra‐subject consistency between the at‐home and in‐clinic assessments, regardless of whether the subject was living with a cat, although the at‐home mean values were slightly lower, and WC subjects had higher variability (Figure 1A). At‐home spirometry resulted in generally similar values at the morning and evening assessments (Figure 1B) and minimal daily variability over the 28 days (data not shown). However, WC subjects manifested greater variability in both morning and evening assessments and mean values that were slightly lower than WoC (Figure 1B). The trend to lower overall average FEV1% predicted measured over the 28 days study period compared to in‐clinic measurements reflects the generally lower FEV1 morning values.

### 3.4 IgG_4_ levels

**FIGURE 2:**
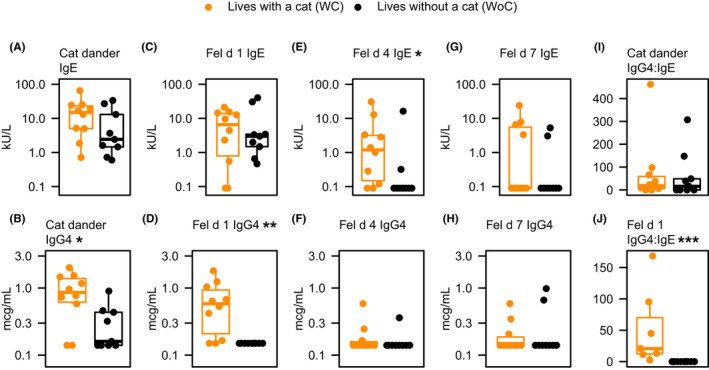
Allergen specific immunoglobulin concentrations are higher in cat‐allergic asthmatics living with cats than in those without cats. *p value <.05, **p value <.01, ***p value <.001 using Mann‐Whitney *U*‐test

In WC subjects, serum IgG_4_ concentrations toward cat dander and Fel d 1 were ~3‐ and 4.5‐fold higher, respectively, than concentrations in WoC subjects (both *p* < .05; Figure 2 and Table S5); measurements were consistent at the two evaluated time points. Fel d 4‐ and Fel d 7‐sIgG_4_ levels were not significantly different between the two groups, likely due to smaller sample size and larger variability of the results. Neither were there significant differences between the two groups for total serum IgE levels, or cat dander‐, Fel d 1‐ or Fel d 7‐sIgE. However, Fel d 4‐sIgE was significantly higher in WC subjects (*p* < .05). In addition, WC subjects had significantly higher Fel d 1 sIgG_4_/sIgE ratio (ng/ml) compared with WoC (*p* < .001), although there was no significant difference between the 2 groups for cat dander sIgG_4_/sIgE ratio (Figure 2 and Table S5). Individual subject data for serum immunoglobulin concentrations are presented in Tables S6 and S7. It is worth noting that allergen‐specific IgE and IgG_4_ measurements on Days 1 and 28 were highly reproducible (Figure S1A). While SPT results were comparable at the two time points (Figure S1B), they were not as reproducible as the serum sIgE measurements; there was no significant difference between the two groups in SPT for standardised cat hair extract (data not shown). Subjects had variable sensitivities to other allergens, but the two groups were not statistically different except for dog allergen (*p* = .02), since there were more dog‐allergic WC than WoC subjects (data not shown).

### 3.5 Basophil sensitivity to Fel d 1

**FIGURE 3:**
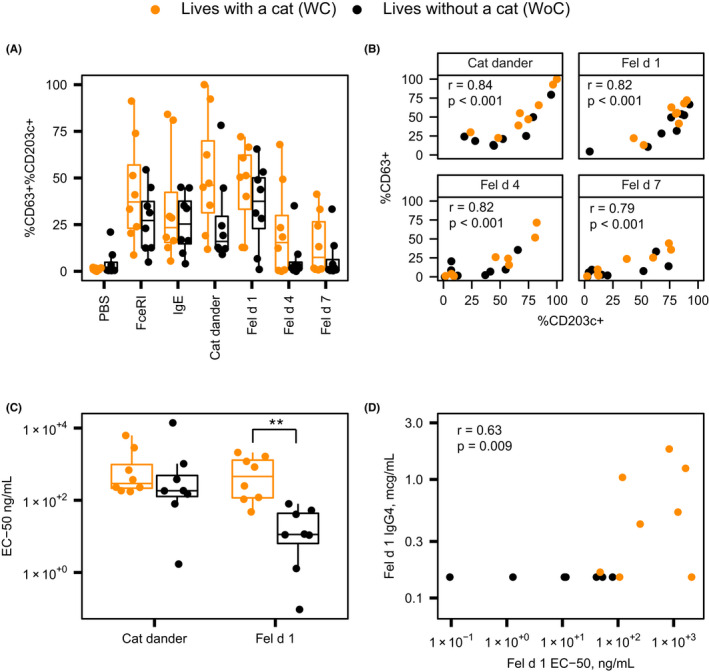
Subjects who live with a cat have lower basophil sensitivity to Fel d 1 which correlates with serum sIgG concentrations. (A) Basophil activation in response to Fel d 1, Fel d 4, and Fel d 7. *PBS*, phosphate buffered saline. (B) Spearman correlation analysis of CD63 expression with expression of CD203c on basophils. (C) Basophil threshold sensitivity (EC_50_) to cat hair extract and Fel d 1. (D) Spearman correlation analysis between Fel d 1 threshold sensitivity (EC_50_) and serum Fel d 1 sIgG_4_ concentration in the total study sample. **p value <.01. Comparison between groups conducted using Mann‐Whitney *U*‐test (panel A) and *t*‐test (panel C)

To further evaluate the impact of daily cat exposure, basophil activation was evaluated *ex vivo* in the presence of cat major allergens (Tables S6 and S7). Two of the 19 cat allergic subjects showed non‐responder basophils (non‐release; 1 WC and 1 WoC) and one WC subject had activated basophils at baseline (persistently active) at all time points and were excluded from the analysis. There were no statistical differences between the two groups in the induction of CD203c and CD63 on basophils following stimulation with 10,000 ng/ml of cat extract (Jubilant HollisterStier Allergy), natural Fel d 1, recombinant Fel d 4 and recombinant Fel d 7 (standard BAT) (Figure 3A and B). Both groups had strong induction of CD203c and CD63 expression following stimulation with natural Fel d 1. However, stimulation with recombinant forms of Fel d 4 and Fel d 7 elicited numerically stronger basophil activation in WC individuals compared to WoC. There were no significant differences between the two groups for BAT reactivity to grass mix and dust mite/dog/mouse mix (data not shown).

Interestingly, CD63 and CD203c expression correlated, suggesting that a certain level of activation (i.e., CD203c) is needed before degranulation (i.e., CD63; Figure 3B). As shown in Figure 3C, WC subjects had significantly decreased basophil sensitivity (higher EC_50_) to cat dander extract and to Fel d 1 compared to WoC subjects, suggesting that people who live with cats require higher concentrations of cat allergens to mount a basophil response. Accordingly, the maximal reactivity was higher in WC subjects compared to the WoC individuals. Furthermore, averaging the parameters of the cat extract basophil activation data showed that while WC subjects had higher reactivity at saturation compared to the WoC individuals, WoC subjects tended to have larger reactions at low concentrations of cat extract (Figure S2). Reduced sensitivity in subjects who lived with cats correlated with accumulation of specific IgG_4_, which has the capacity to attenuate IgE mediated allergic symptoms (Figure 3D). Importantly, longitudinal consistency was observed for CD63 and EC_50_ values (Figure S3), indicating robustness of the findings.

### 3.6 T‐cell reactivity to Fel d 1 and Fel d 4

**FIGURE 4:**
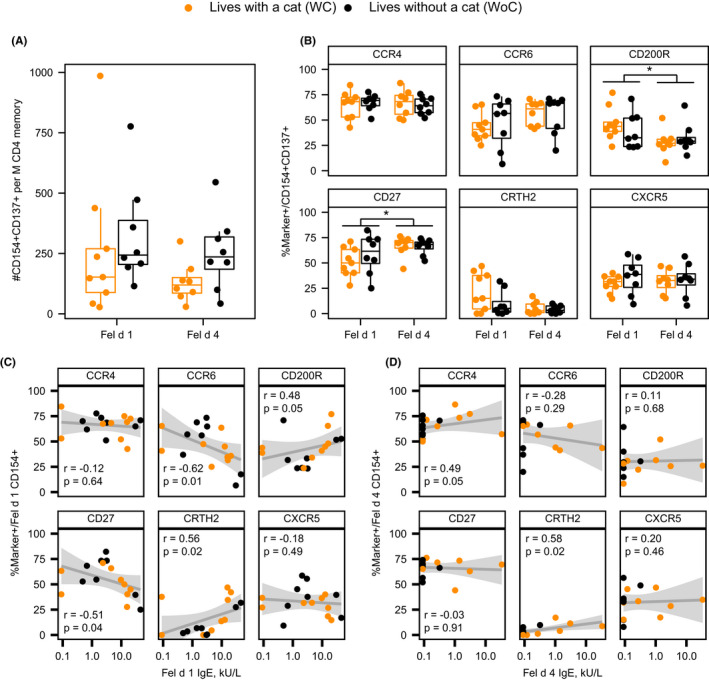
Fel d 1 elicits a greater TH2A‐cell immune response than Fel d 4. **(**A) Frequency of cat‐reactive T‐cells elicited by natural Fel d 1 or recombinant Fel d 4. (B) Expression of CD154+ T‐cell marker subsets. (C) Correlation between CD154+ T‐cell markers and Fel d 1‐specific IgE. (D) Correlation between CD154+ T‐cell markers and Fel d 4‐specific IgE. Grey areas in C and D represent the 95% confidence bands for a linear fit. Mann‐Whitney *U*‐test conducted for comparisons between groups (panels A and B) as well as for comparison between Fel d 1 and Fel d 4 (panel B). Spearman correlation coefficients are presented in panels C and D

CD154/CD137 up‐regulation assay was used to assess Fel d 1 and Fel d 4‐reactive CD4+ T effector (cTeff) and T‐regulatory (cTreg) cell responses. WoC subjects tended to have higher frequencies of cTeff and cTreg responses to Fel d 1 and Fel d 4, but the differences between groups were not statistically significant (Figure 4A and Figure S4). While Fel d 1 exposure trended toward towards a Th2 phenotype in WC subjects and Th17 in WoC subjects, there was no difference between WC and WoC for Fel d 4 exposure (Figure 4B). Interestingly, we observed moderate‐to‐strong significant positive correlations between surface expression of CRTH2 and CD200R on Fel d 1‐reactive T cells and the level of serum Fel d 1 specific IgE (*r* = 0.56; *p* = .02 and *r* = 0.48; *p* = .05, respectively; Figure 4C). In contrast, expression of CD27 and of CCR6 on surface of Fel d 1‐reactive T cells showed a strong negative correlation with serum Fel d 1 sIgE (*r* = −0.51; *p* = .04 and *r* = −0.62; *p* = .01, respectively), but no correlation with Fel d 4 sIgE (Figure 4D). Similarly, a strong and significant positive correlation was observed between CRTH2 expression on Fel d 4‐reactive T cells and serum Fel d 4 specific IgE (*r* = 0.58; *p* = .02; Figure 4D). No correlation was observed between basophil reactivity and T‐cell responses against individual cat allergens (data not shown).

### 3.7 Cytokine profiles

To further investigate differences between Fel d 1‐ and Fel d 4‐specific CD4+ T cell responses, a direct comparison of the cytokine profiles was conducted using in vitro staining for intracellular cytokines and MHCII tetramers in a separate cohort of cat‐allergic subjects (N = 10; 2 WC, 6 WoC, and 2 unknown) with DR04:01 or DR15:01 haplotypes. These subjects, who were recruited at the allergy clinic at Virginia Mason Medical Center with written consent as part of an IRB‐approved study, were selected based on their clinical symptoms, a positive SPT, and serum IgE response to cat dander >0.35 kU_A_/ml using the ImmunoCap test (Phadia AB). We selected DRB1*04:01 and HLA‐DRB1*15:01‐DRB5*01:01 (DR15) haplotypes as these alleles were prevalent in our cohort and immunodominant Fel d 1 and Fel d 4‐derived peptides were previously described for these alleles.^8^, ^23^ Fel d 1 responses in cat allergic individuals were associated with strong IL‐4‐ and IL‐5‐producing CD4^+^ T cells (Th2A profile). Conversely, in these individuals, Fel d 4 elicited dominant IFN‐γ‐producing CD4^+^ T cells (Th1 profile) and a moderate IL‐4 response in the absence of IL‐5 (Figure S5). Interestingly, co‐staining for cytokines in Fel d 4 tetramer‐positive cells showed that IL‐4‐producing cells were mutually exclusive from IFN‐γ‐producing CD4^+^ T cells (Figure S5).

## 4 DISCUSSION

This observational non‐interventional study explored how clinical and immunological characteristics related to cat allergy differ between asthmatic subjects who live with cats and those who do not, albeit in a low number of subjects. We demonstrated that chronic cat allergen exposure modulates the relationship between primary effector cells (basophils) and cat allergen‐specific IgG_4_. A unique aspect of the study is that such cohorts have not previously been comprehensively evaluated. In addition, clinical and immunological outcomes were evaluated at serial time points over a 28 days period. Basophil activation was assessed both qualitatively and quantitatively, and the induction of cat allergen‐specific Th2A cells was specifically evaluated. Of particular relevance is that the time points provided evidence of within‐subject and longitudinal robustness of BAT and BST as immunological markers, showing a consistency similar to SPT and serum testing. Additionally, five subjects who had negative Fel d 4 sIgE and seven subjects with negative Fel d 7 sIgE had positive BATs to Fel d 4 and Fel d 7, respectively, suggesting that basophil testing is more sensitive than serology as a marker for allergy, and its use as an outcome measure should be further explored as a potential surrogate of treatment effects.^24^

In both groups of cat allergic individuals, Fel d 1 was the most prevalent cat allergen leading to high serum sIgE levels and significant basophil reactivity. However, WC subjects tended to have higher sIgE levels and basophil reactivity to Fel d 1 compared to WoC, which may result from the continued exposure to additional epitopes on Fel d 1. Chronic exposure to cats might also contribute to sensitisation to other cat allergen components, as we observed significantly higher levels of sIgE against Fel d 4, along with numerically greater basophil reactivity to Fel d 4 and Fel d 7, among WC relative to WoC subjects.

Increased IgG_4_ has previously been implicated in ameliorating the IgE‐mediated allergic response and in inducing tolerance.^8^ Beyond IgG_4_, it has been further suggested that natural cat exposure results in partial clinical and immunological tolerance that may be mediated by other subclasses of IgG, or more specifically by the relative abundance of IgG vs. IgE.^7^ The high IgG concentrations obtained with AIT or during passive administration of monoclonal antibodies also increase the IgG/IgE ratio, which has been reported to correlate with clinical symptom improvement.^9^, ^25^ Consistent with these studies, we observed that living with a cat was associated with increased Fel d 1 sIgG_4_ levels, which correlated with significantly lower basophil sensitivity to cat dander extract and Fel d 1 protein relative to WoC subjects.

Despite the presence of sIgG_4_, WC subjects still experienced nasal, ocular, and respiratory symptoms that required medication use. The sIgG_4_ concentrations in naturally exposed WC subjects were substantially lower than those achieved during cat specific AIT, or by passive administration of allergen‐specific IgG_4_ monoclonal antibodies, both treatments shown to provide symptomatic benefits in cat‐allergic subjects.^9^, ^25^, ^26^ AIT has been shown to induce other allergen‐specific IgG subclasses (including IgG_1_, IgG_2_ and IgG_3_),^9^ thus IgG_4_ may not be the only type of immunoglobulin that is functionally protective.^27^

The similar lung function, nasal patency, rhinitis symptoms and asthma control between WC and WoC subjects may be explained, at least in part, by the higher medication burden among the WC subjects. This medication burden may enable symptomatic control, since continuous natural cat exposure does not sufficiently attenuate the allergic response. There remains a substantial unmet need for more effective management strategies in subjects with cat allergy/asthma who are living with cats. Furthermore, in trials of such management strategies, use of lung function as an endpoint was supported by the consistency between at‐home and in‐clinic spirometry assessments.

Interestingly, BST showed that at least a 50% level of CD203c activation is needed to trigger CD63 expression (which reflects histamine release) in these cat‐allergic subjects. Although all subjects had positive basophil response to natural Fel d 1, WC subjects required higher concentration of natural Fel d 1 to trigger basophil activation. Basophil sensitivity (EC_50_) for Fel d 1 showed a strong correlation with serum Fel d 1 sIgG_4_ concentration, which may be due to the blocking activity of sIgG_4_ to Fel d 1. Although WC subjects are less sensitive to Fel d 1 stimulation (EC_50_), their basophil reactivity, as measured by CD203c and CD63 expression to Fel d 1, as well as to other cat allergens (Fel d 4 and 7), was stronger when compared to WoC. Higher basophil or mast cell reactivity to Fel d 1 may be one of mechanisms underlying the stronger requirement for medication use observed in WC subjects. While it is possible that mast cell desensitisation can be produced without a similar effect in basophils,^28^, ^29^ the presence of differences could not be evaluated and represents a limitation of the current study. CD27 expression has been shown to determine the pathogenic relevance of allergen‐specific T cells and to reflect repeated antigen exposure.^30^, ^31^, ^32^ In this study, CD27 expression in cat allergen‐reactive CD4+ T cells do not distinguishes cat allergic WC individuals from WoC individuals. However, prolonged exposure to cat allergen may have contributed to the decreased CD27 expression and strong Th2A response against Fel d 1 compared to Fel d 4, despite eliciting strong IgE response to both antigens. Use of tetramer based‐T cell assays showed that Fel d 1 specific Th2A cells are high producers of IL‐4, L‐5 and IL9, and low producers of IFNγ. These data indicate that Fel d 1 as the dominant allergen cat component and may be playing a key role in driving the Th2‐cell mediated allergic response. Paradoxically, Fel d 1‐ and Fel d 4‐specific CD4+ T‐cell responses in WoC individuals were not differentially polarised and tended to have higher expression of the Th17‐related marker CCR6 and the TFH‐related marker, CXCR5.

## 5 CONCLUSIONS

Cat allergen exposure modulates the relationship between primary effector basophils and allergen‐specific blocking IgG_4_, with Fel d 1 appearing to be the primary allergen. Even though we observed immunological changes in WC subjects relative to WoC subjects, the clinical results do not support tolerance with natural cat exposure. Despite the small sample size, which is the primary limitation of this study, the results showed low intra‐subject variability and remarkable robustness in longitudinal measurement of clinical assessments and immunological assays. We also observed immunological differences that could distinguish between the two groups with statistical significance. These immunological differences suggest the importance of designing studies for evaluation of allergy management strategies that specifically reflect the population of interest and their baseline clinical/immunological characteristics. The data also imply a need to stratify the cat allergic population based on living with a cat. Finally, the clinical and immunological markers that we evaluated in this study should be further explored as surrogate measure of therapeutic effects in future clinical trials, and also as biomarkers for mechanistic studies of the allergic response.

## CONFLICT OF INTEREST

M Ruddy, M DeVeaux, P Meier, CQ Wang, and A Radin are employees and shareholders of Regeneron Pharmaceuticals.

## AUTHOR CONTRIBUTIONS

E.R. Wambre, M. Farrington, H.A. DeBerg, M. DeVeaux, C.Q. Wang and A. Radin contributed in the experimental design, data analysis, manuscript writing, review and editing; V. Bajzik contributed immune mechanism investigation and D. Robinson, M. Farrington, M. Cantor and C. Huang were involved in clinical investigation and data analysis; P. Meier provided project administration; M. Ruddy and J.M. Orengo contributed design review and manuscript review and editing.

## ETHICAL APPROVAL

The study received Institutional Review Board approval (Benaroya Research Institute Institutional Review Board; IRB07109‐519), and all subjects provided written informed consent and Health Insurance Portability and Accountability Act (HIPAA) authorisation prior to participation.

## Supporting information

Supplementary Material

## Data Availability

The data that support the findings of this study are available from the corresponding author upon reasonable request.
