# Supplementary material for: Clinical and immunological evaluation of cat‐allergic asthmatics living with or without a cat
Source: Clin Exp Allergy. 2021 Oct 8;51(12):1624–33. doi: 10.1111/cea.14024 (PMC9293312; doi:10.1111/cea.14024)
Supplement: Supplementary file 1 — Supplementary Material [file CEA-51-1624-s001.docx]

**ONLINE SUPPLEMENTARY MATERIAL**

**Clinical and Immunological Evaluation of Cat-allergic Asthmatics Living With or Without a Cat**

Erik R. Wambre, Mary Farrington, Veronique Bajzik, Hannah A. DeBerg, Marcella Ruddy, Michelle DeVeaux, Pretty Meier, David Robinson, Matt Cantor, Chengrui Huang, Claire Q. Wang, Allen Radin

**Supplementary Methods**

*Spirometry*

Forced expiratory volume in 1 second (FEV1) was assessed at home mornings and evenings using a hand-held respiratory device provided by Koneksa Health (New York, NY, USA), which consisted of a smartphone application and Bluetooth enabled mobile spirometer, mSpirometer®, and the BreatheSmart®. The application guided the subjects through the spirometry assessments and results were collected in the Koneksa Health Compare portal. FEV1 was also measured on Days 1 and 28 during the in-clinic visit using the same device as for home assessment as well as with a standard in-clinic spirometer (KOKO SX1000 Hand-Held Spirometer/ Computer Based, Nspire Health, Inc., Longmont, CO, USA). The peak nasal inspiratory flow (PNIF) was assessed at the Days 1 and 28 in-clinic visits using an In-Check portable nasal inspiratory flow meter (Clement Clarke International, Harlow, Essex, UK).

*Subject-reported measures of clinical symptoms*

The Total Nasal Symptom Score (TNSS)^1^ was modified for a 12-hour recall period and separated itching and sneezing as individual items, with all 4 items scored from 0 = none to 3 = severe. The Total Ocular Symptom Score (TOSS) was based on 2 items (eyes itching/burning, and eyes watering/tearing) scored from 0 = none to 3 = severe using a 12-hour recall period. The Asthma Control Questionnaire (ACQ)^2^ has a 1-week recall period, with 6 items scored using a 7-point Likert scale that generates a global score (higher score = poorer control). The Rhinoconjunctivitis Quality of Life Questionnaire (RQLQ)^3^ has a 1-week recall period and consists of 28 questions scored on a 7-point Likert scale that generates a global score and 7 domain scores (higher scores = poorer health-related quality of life); the minimum clinically important difference (MCID) on the global score has been estimated at 0.5.^4^ TNSS and TOSS assessments were obtained using the study-provided smartphone using a Koneksa Health eDiary application with data collected in the Koneksa Health Compare portal. ACQ and RQLQ assessments were obtained with paper questionnaires provided in a study binder.

*Skin prick test*

The SPT (Jubilant HollisterStier Allergy, Spokane, WA , USA), conducted via a standard protocol,^5^ used standardized extracts for cat hair (10,000 BAU/mL), as well as mouse extract (1:20 dilution), dust mite extracts (both *Dermatophagoides farinae* [DMF] and *Dermatophagoides pteronyssinus* [DMP]; 10,000 AU/mL), acetone-precipitated (AP) dog hair and dander extract (100,000 BAU/mL), 7 grass mix (Kentucky Blue/June, Meadow Fescue, Orchard, Perennial Ryegrass, Redtop, Sweet Vernal, Timothy; 100,000 BAU/mL) and both negative (sodium chloride 0.9%) and positive controls (10 mg/mL histamine phosphate 2.75 mg/mL, glycerin 50%).

*Ex vivo analysis of cat allergen-reactive CD4+ T cells*

Cat allergen-reactive CD4+ T cells were tracked using the CD154 up-regulation assay.^6,7^. Briefly, 10-20 x 10^6^ freshly isolated PBMCs in culture medium (10 x 10^6^ cells/mL) were stimulated with a pooled library of 20-mers peptides derived from Fel d 1 or Fel d 4 cat allergen and 1 µg/mL of anti-CD40 blocking mAb (clone HB14; Miltenyi Biotec, Auburn, CA). After 18 hours stimulation at 37°C, cells were harvested and labeled with PE-Conjugated anti-CD154 mAb for 10 minutes at 4°C. Cells were then washed, labeled with anti-PE magnetic beads and enriched by using a magnetic column, according to the manufacturer’s instructions (Miltenyi Biotec, Bergisch Gladbach, Germany). Magnetically enriched cells were stained with antibodies against markers of interest and analyzed on a FACSAria™ II flow cytometer (BD Biosciences, San Jose, CA). Live memory CD45RA- CD154+ CD4+ T cells were considered cat-reactive CD4+ T effector cells. A combination of the vital dye Via-Probe (BD Pharmingen) as a viability marker, CD19 (eBioscience), and CD14 (eBioscience) was used to exclude dead cells, B cells, and monocytes from the analysis, respectively. Frequency was calculated as previously described.^8^ Data were analyzed with FlowJo software (Tree Star, Inc., Ashland, OR).

**Supplementary Figures and Tables**

**FIGURE S1** Correlations between days 1 and 28 show high rates of reproducibility of assessment for IgE (A) and skin prick test (B). Pearson’s correlation coefficients are reported (r).


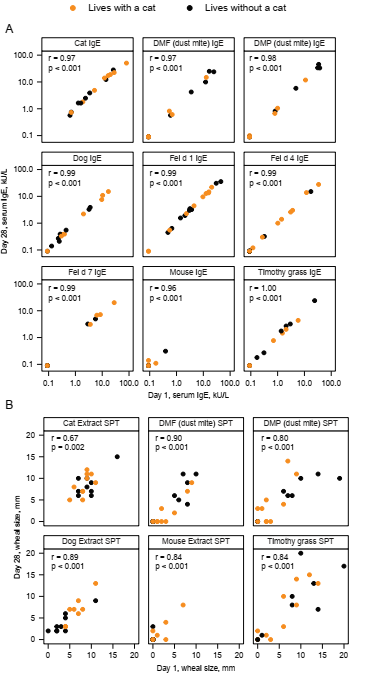


**FIGURE S2** Average basophil reactivity dose-response curves to cat extract.
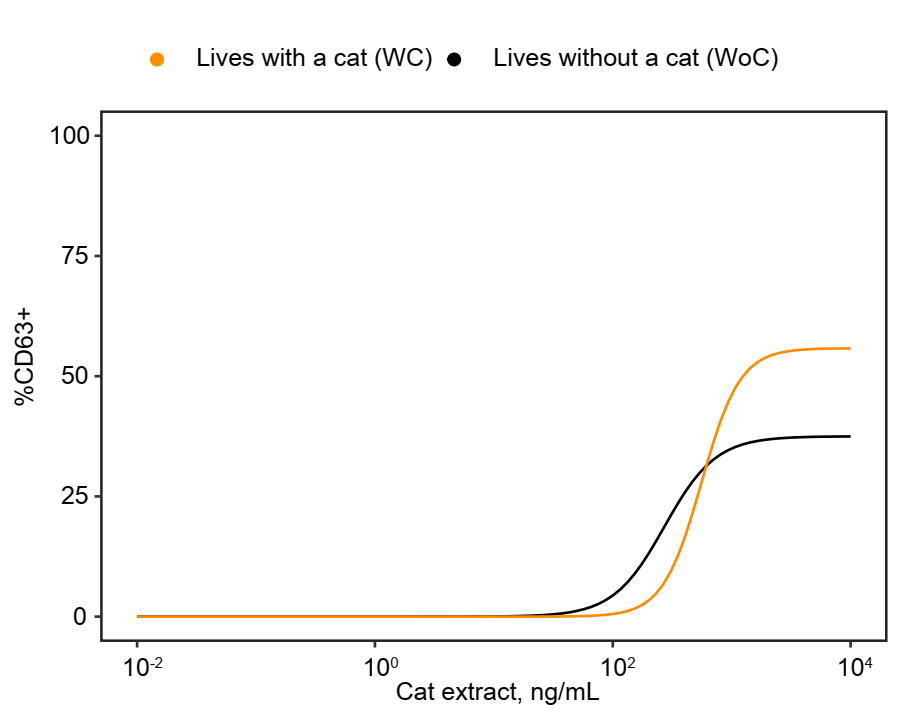


**FIGURE S3** Longitudinal consistency in the basophil activation assay. A) Strong correlation of basophil activation after *ex vivo* allergen stimulation on Day 1 and Day 28 in the total study population. B) Reproducibility of the basophil sensitivity test to Fel d 1 on Days 1 and 28 in a representative cat allergic subject. Pearson’s correlation coefficients are reported (r).


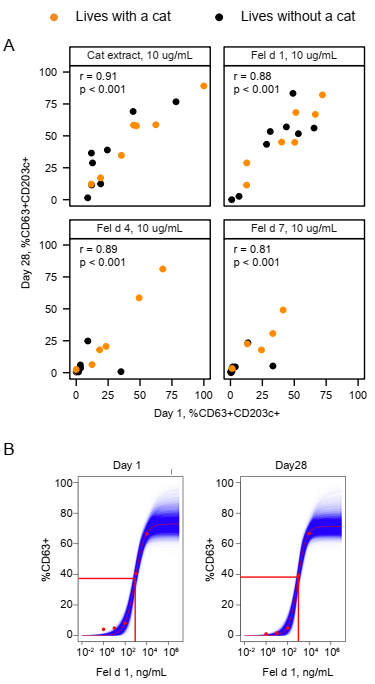


**FIGURE S4** Frequency of Fel d 1- and Fel d 4-reactive T-regulatory (cTreg) cells per million total CD4+ cells. Fel d 1- and Fel d 4-reactive cTreg cells were evaluated using a CD154/CD137 up-regulation assay. Comparison between groups conducted using Mann-Whitney U-test.


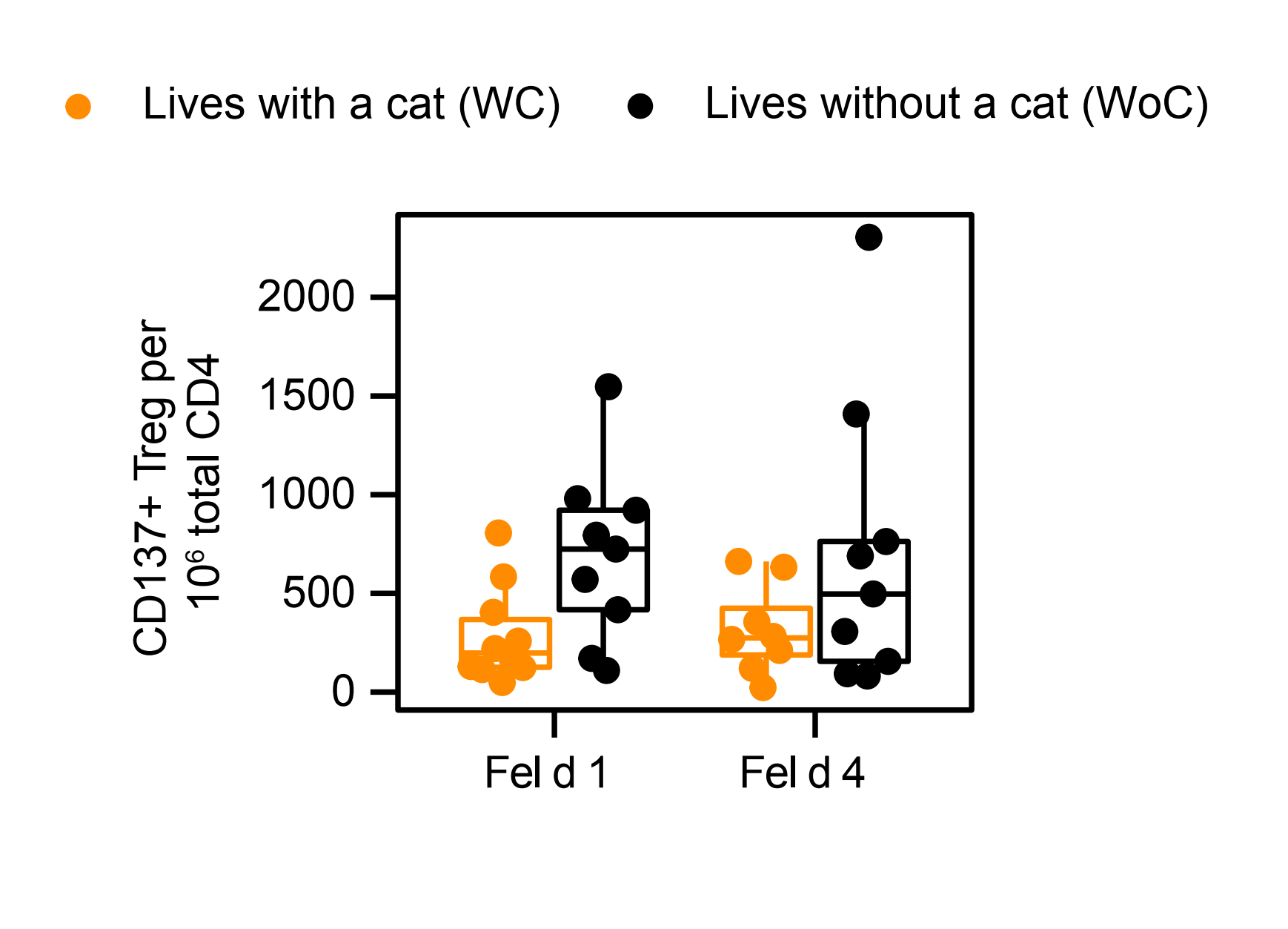


**FIGURE S5** Flow cytometry evaluating cytokine expression in T cells from cat allergic individuals. Representative plots showing cytokine expression of (**A**) Fel d 1- and (**B**) Fel d 4-specific T cells in DR04:01-restricted cells in cat allergic individuals. Percentages of tetramer-positive cells expressing the given marker are indicated in the upper right quadrant. Representative flow cytometry plots showing co-production of IFN-γ and IL-4 by (**C**) Fel d 1- or (**D**) Fel d 4-specific CD4+ T cells from cat allergic individuals. **A-D**, Data are representative for at least 5 individuals per group. (**E**) Cytokine production by Fel d 1- and Fel d 4-specific T cells in cat allergic individuals. Data are mean ± standard error from at least 5 individuals. Differences between groups were analyzed by Mann-Whitney U-test. ***P* value < .01, ****P* value < .001.


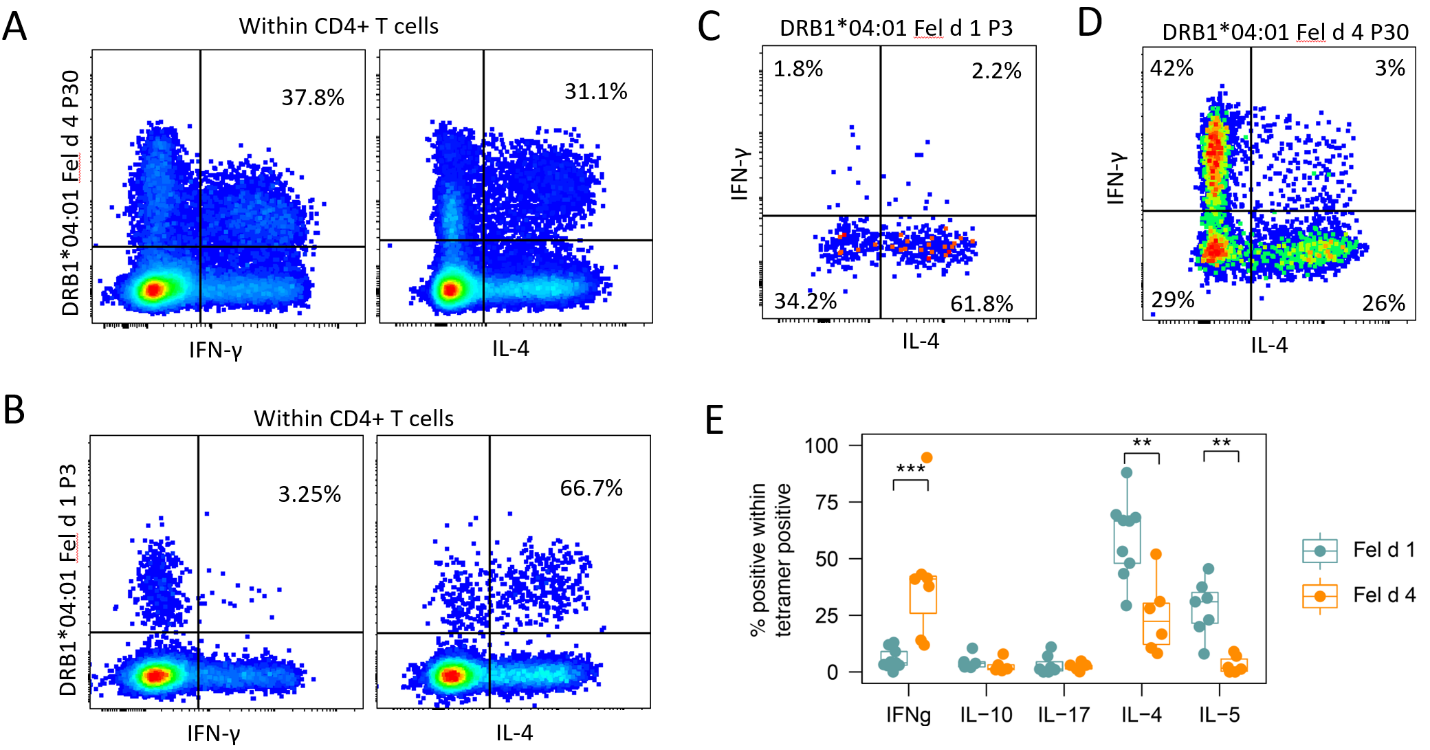


**TABLE S1** Demographic characteristics of the study population

| **Variable** | **Lives with a cat**  **(n = 10)** | **Does not live with a cat**  **(n = 9)** |
| --- | --- | --- |
| Age, y, mean ±SD | 42.3 ± 9.9 | 38.4 ± 12.6 |
| Sex, no. (%) |  |  |
| Male | 5 (50.0) | 4 (44.4) |
| Female | 5 (50.0) | 5 (55.6) |
| Race, no. (%) |  |  |
| White | 9 (90) | 8 (88.9) |
| Asian | 1 (10) | 1 (11.1) |
| Ethnicity, no. (%) |  |  |
| Hispanic or Latino | 1 (10.0) | 0 |
| Not Hispanic or Latino | 9 (90.0) | 9 (100) |
| Not reported | 0 | 0 |
| Height, cm, mean ± SD | 170.4 ± 12.8 | 169.8 ± 11.0 |
| Weight, kg, mean ± SD | 89.8 ± 20.9 | 85.7 ± 27.8 |
| Body mass index, kg/m^2^, mean ± SD | 30.7 ± 4.9 | 29.7 ± 9.3 |
| Dog ownership, n (%) | 1 (10) | 4 (44.4) |

**TABLE S2** Details of subjects living with cats

| **Subject** | **Number of cats** | **Length of time living with cat(s), months** | **Cats allowed in bedroom** |
| --- | --- | --- | --- |
| 1 | 2 | 72 | Yes |
| 2 | 2 | 98 | Yes |
| 3^a^ | 1 | 18 | No |
| 4 | 10 | 144 | No |
| 5 | 1 | 204 | Yes |
| 6 | 1 | 132 | No |
| 7 | 1 | 60 | Yes |
| 8 | 2 | 36 | Yes |
| 9 | 1 | 222 | Yes |
| 10 | 2 | 132 | Yes |

^a^Dog in home

**TABLE S3** Home allergen exposure measured from bedroom dust

| **Allergen** | **mcg/g bedroom dust, median (range)** | |
| --- | --- | --- |
|  | **Lives with a cat**  **(n = 10)** | **Does not live with a cat**  **(n = 9)** |
| Fel d 1 (cat) | 37.5 (0.6-392.6) | 0.1 (0.02-2.5) |
| Fel d 4 (cat) | 10.4 (0.09-266.2) | 0 (0-0.2) |
| Can f 1 (dog) | 0.04 (0-108.0) | 1.7 (0.07-147.2) |
| Der f 1 (dust mite) | 0 (0-0.03) | 0 (0-0.7) |
| Der p 1 (dust mite) | 0 (0-0.3) | 0 (0-0.1) |
| Mus m 1 (mouse) | 0 (0-0) | 0.001 (0-0.004) |
| Phl p 5 (Timothy grass) | 0.02 (0-0.1) | 0 (0-0.6) |

**TABLE S4** Clinical measures

| **Variable** | **Lives with a cat**  **(n = 10)** | **Does not live with**  **a cat (n = 9)** |
| --- | --- | --- |
| FEV1, L, mean ± SD |  |  |
| In-clinic | 2.7 ± 0.8 | 2.9 ± 0.7 |
| Home 28-day average | 2.5 ± 0.8 | 2.8 ± 0.7 |
| FEV1 % predicted, mean ± SD |  |  |
| In-clinic | 78.0 ± 20.5 | 80.8 ± 15.8 |
| Home 28-day average | 72.2 ± 21.2 | 76.5 ± 16.4 |
| PNIF, L/min, mean ± SD |  |  |
| Males (normal > 175) | 147.1 ± 50.3 | 138.6 ± 58.4 |
| Females (normal > 125) | 111.2 ± 28.6 | 126.9 ± 35.2 |
| TNSS, mean ± SD | 2.4 ± 1.3 | 1.8 ± 1.8 |
| TOSS, mean ± SD | 1.4 ± 0.6 | 0.7 ± 0.9 |
| DMS, mean ± SD | 4.0 ± 0.7* | 2.5 ± 1.8 |
| CSMS, mean ± SD | 7.8 ± 2.2* | 5.0 ± 2.3 |
| Asthma severity, no. (%) |  |  |
| Mild intermittent | 0 | 2 (22.2) |
| Mild persistent | 1 (10.0) | 3 (33.3) |
| Moderate persistent | 8 (80.0) | 3 (33.3) |
| Severe persistent | 1 (10.0) | 1 (11.1) |
| Use of long-acting bronchodilators, no. (%) | 9 (90.0)* | 2 (22.2) |
| Use of inhaled corticosteroids, no. (%) | 9 (90.0) | 6 (66.7) |
| Daily average inhaled corticosteroids, µg, mean ± SD | 489.0 ± 292.8 | 262.2 ± 279.4 |
| ACQ, mean ± SD | 1.3 ± 0.7 | 1.0 ± 0.9 |
| RQLQ, mean ± SD | 2.1 ± 0.9* | 1.0 ± 0.8 |

*ACQ*, Asthma Control Questionnaire; *CSMS*, Combined Symptom and Medication Score; DMS, Daily Medication Score; *FEV1*, forced expiratory volume in one second; *PNIF*, Peak Nasal Inspiratory Flow; *RQLQ*, Rhinoconjunctivitis Quality of Life Questionnaire; *TNSS*, Total Nasal Symptom Score; *TOSS*, Total Nasal Symptom Score

**P* value < .05 vs does not live with a cat

**TABLE S5** Serum antibody concentrations averaged from assessments on Days 1 and 28

| **Variable** | **Mean ± SD** | |
| --- | --- | --- |
|  | **Lives with a cat**  **(n = 10)** | **Does not live with**  **a cat (n = 9)** |
| Cat dander IgG_4_, mcg/mL | 0.95 ± 0.6* | 0.32 ± 0.3 |
| Fel d 1 IgG_4_, mcg/mL | 0.68 ± 0.5* | 0.15 ± 0.0 |
| Fel d 2 IgG_4_, mcg/mL | 0.21 ± 0.1 | 0.15 ± 0.02 |
| Fel d 4 IgG_4_, mcg/mL | 0.20 ± 0.1 | 0.16 ± 0.07 |
| Fel d 7 IgG_4_, mcg/mL | 0.17 ± 0.17 | 0.24 ± 0.34 |
| Total IgE, kU/L | 204.7 ± 124,5 | 195.7 ± 131.1 |
| Cat dander IgE, kU/L | 18.1 ± 18.6 | 9.4 ± 12.8 |
| Fel d 1 IgE, kU/L | 8.1 ± 7.6 | 9.5 ± 15.0 |
| Fel d 2 IgE, kU/L | 0.55 ± 0.9 | 0.10 ± 0.01 |
| Fel d 4 IgE, kU/L | 5.3 ± 9.8* | 1.9 ± 5.4 |
| Fel d 7 IgE, kU/L | 4.2 ± 7.6 | 0.97 ± 1.9 |
| Fel d 1 IgG_4_/IgE ratio | 35.6 ± 52.4* | 0 |
| Cat dander IgG_4_/IgE ratio | 71.8 ± 133.7 | 61.9 ± 97.5 |

**P* value < .05 vs does not live with a cat

**Table S6** Cat dander- and Fel d 1-specific measures in individual subjects

| **Subject** | **Cat dander sIgE, kU/L** | **cat dander sIgG_4_, mcg/mL** | **Cat dander IgG_4_/ IgE ratio, ng/mL** | **Fel d 1 sIgE, kU/L** | **Fel d 1 sIgG_4_, mcg/mL** | **Fel d 1 IgG_4_/ IgE ratio, ng/mL** | **Total sIgE, kU/L** | **Positive cat dander BAT,** **10,000 ng/mL** | **Positive nFel d 1 BAT, 10,000 ng/mL** |
| --- | --- | --- | --- | --- | --- | --- | --- | --- | --- |
| Lives with a cat |  |  |  |  |  |  |  |  |  |
| 1 | 16.8 | 1.54 | 38.20 | 12.8 | 0.64 | 20.8 | 820 | —^a^ | —^a^ |
| 2 | 63.1 | 0.96 | 6.30 | 21.3 | 0.68 | 13.3 | 383 | —^b^ | —^b^ |
| 3^c^ | 22.3 | 0.77 | 14.40 | 15.4 | 0.42 | 11.4 | 215 | Yes | Yes |
| 4 | 1.8 | Negative^d^ | — | 0 | Negative | — | 27 | Yes | Yes |
| 5 | 25.7 | 0.58 | 9.40 | 14.7 | 0.09 | 2.6 | 285 | Yes | Yes |
| 6 | 5.68 | Negative | — | 0.56 | Negative | — | 69 | Yes | Yes |
| 7 | 26.9 | 1.47 | 22.80 | 9.65 | 1.04 | 44.9 | 318 | Yes | Yes |
| 8 | 4.95 | 1.17 | 98.30 | 2.32 | 0.53 | 95.2 | 183 | Yes | Yes |
| 9 | 12.7 | 2.01 | 65.90 | 4.48 | 1.81 | 168.3 | 168 | Yes | Yes |
| 10 | 0.68 | 0.74 | 462.50 | 0 | 1.24 | — | 31 | Yes | Yes |
| Does not live with a cat |  |  |  |  |  |  |  |  |  |
| A^c^ | 12.3 | 0.46 | 15.60 | 3.44 | Negative | — | 256 | Yes | Yes |
| B^c^ | 0.58 | 0.43 | 307.1 | 0.47 | Negative | — | 11 | Yes | Yes |
| C | 27 | Negative | — | 30.5 | Negative | — | 281 | Yes | Yes |
| D | 1.44 | 0.17 | 48.6 | 1.49 | Negative | — | 48 | Yes | Yes |
| E | 34.6 | Negative | — | 40.3 | Negative | — | 124 | Yes | Yes |
| F^c^ | 0.72 | Negative | — | 0.66 | Negative | — | 389 | Yes | Yes |
| G | 3.46 | 0.32 | 38.6 | 3.33 | Negative | — | 214 | Yes | Yes |
| H^c^ | 1.48 | Negative | — | 2 | Negative | — | 56 | Yes | Yes |
| I | 2.55 | 0.9 | 147.5 | 3.01 | Negative | — | 611 | —b | —b |

^a^Non-responsive basophils

^b^Persistently active basophils

^c^Dog in home

^d^Values ≤ 0.15 mcg/mL were considered negative.

**Table S7** Fel d 4- and Fel d 7-specific measures in individual subjects

| **Subject** | **rFel d 4 kU/L** | **rFel d 4 sIgG4, mcg/mL** | **rFel d 7 sIgE, kU/L** | **rFel d 7 sIgG4, mcg/mL** | **Positive rFel d 4 BAT, 10,000 ng/mL** | **Positive rFel d 7 BAT,** **10,000 ng/mL** |
| --- | --- | --- | --- | --- | --- | --- |
| Lives with a cat |  |  |  |  |  |  |
| 1 | 0.12 | 0.25 | Negative^a^ | Negative | —b | —b |
| 2 | 1.4 | Negative | Negative | Negative | —c | —c |
| 3^d^ | Negative | Negative | Negative | Negative | Yes | No |
| 4 | 1.1 | Negative | Negative | Negative | Yes | Yes |
| 5 | 2.83 | Negative | 7.86 | 0.17 | Yes | Yes |
| 6 | 12.85 | Negative | 6.58 | neg | Yes | Yes |
| 7 | 30.8 | 0.17 | 24 | 0.35 | Yes | Yes |
| 8 | 0.29 | Negative | Negative | Negative | Yes | Yes |
| 9 | 3.32 | 0.59 | 3.35 | 0.59 | Yes | Yes |
| 10 | Negative | Negative | Negative | Negative | Yes | Yes |
| Does not live with a cat |  |  |  |  |  |  |
| A^d^ | 16.25 | 0.36 | 5.29 | 0.98 | No | Yes |
| B^d^ | Negative | Negative | Negative | 0.66 | Yes | Yes |
| C | Negative | Negative | Negative | Negative | Yes | Yes |
| D | Negative | Negative | Negative | Negative | No | No |
| E | Negative | Negative | 3.06 | Negative | Yes | Yes |
| F^b^ | Negative | Negative | Negative | Negative | Yes | Yes |
| G | Negative | Negative | Negative | Negative | Yes | Yes |
| H^b^ | 0.32 | Negative | Negative | Negative | Yes | Yes |
| I | Negative | Negative | Negative | Negative | —^c^ | —^c^ |

^a^Values ≤ 0.15 mcg/mL were considered negative.

^b^Non-responsive basophils

^c^Persistently active basophils

^d^Dog in home

**Supplementary References**

1. Downie SR, Andersson M, Rimmer J, et al. Symptoms of persistent allergic rhinitis during a full calendar year in house dust mite-sensitive subjects. *Allergy.* 2004;59(4):406-414.

2. Juniper EF, O'Byrne PM, Guyatt GH, Ferrie PJ, King DR. Development and validation of a questionnaire to measure asthma control. *Eur Respir J.* 1999;14(4):902-907.

3. Juniper EF, Guyatt GH. Development and testing of a new measure of health status for clinical trials in rhinoconjunctivitis. *Clin Exp Allergy.* 1991;21(1):77-83.

4. Juniper EF, Guyatt GH, Griffith LE, Ferrie PJ. Interpretation of rhinoconjunctivitis quality of life questionnaire data. *J Allergy Clin Immunol.* 1996;98(4):843-845.

5. Bernstein IL, Li JT, Bernstein DI, et al. Allergy diagnostic testing: an updated practice parameter. *Ann Allergy Asthma Immunol.* 2008;100(3 Suppl 3):S1-148.

6. Frentsch M, Arbach O, Kirchhoff D, et al. Direct access to CD4+ T cells specific for defined antigens according to CD154 expression. *Nat Med.* 2005;11(10):1118-1124.

7. Chattopadhyay PK, Yu J, Roederer M. A live-cell assay to detect antigen-specific CD4+ T cells with diverse cytokine profiles. *Nat Med.* 2005;11(10):1113-1117.

8. Kwok WW, Roti M, Delong JH, et al. Direct ex vivo analysis of allergen-specific CD4+ T cells. *J Allergy Clin Immunol.* 2010;125(6):1407-1409 e1401.
